# Application of a three-dimensional virtual model to study the effect of fluoroscopic angle on infra-acetabular corridor parameters and screw insertion rates

**DOI:** 10.1186/s13018-021-02730-w

**Published:** 2021-09-26

**Authors:** Nengfeng Ma, Xufeng Hu, Zhoushan Tao, Min Yang

**Affiliations:** Department of Orthopaedics Trauma, Yijishan Hospital, Wannan Medical College, No.2 Zheshan West Road, Wuhu, 241001 Anhui China

**Keywords:** Infra-acetabular corridor, Infra-acetabular screw, Acetabular fracture, Asian population

## Abstract

**Purpose:**

To use three-dimensional (3D) virtual models to study how the parameters and insertion rates of the infra-acetabular corridor (IAC) change under different fluoroscopic angles.

**Methods:**

The pelvis computed tomography data of 187 patients are imported into Mimics software in DICOM format to generate a 3D model. The anterior pelvis plane is used as the reference plane to measure the diameter of the optimum IAC when the pelvis model is tilted forward by 5°, 15°, 25°, 35° and 45°. The diameter of at least 3.5 mm is defined as the cutoff for placing a 3.5 mm screw, the rate of infra-acetabular screw (IAS) insertion is calculated, and the mean length of the IAC and the mean tilt of the corridor axis in relation to the sagittal midline plane (SMP) are measured.

**Results:**

The similar diameters of the IAC can be found under fluoroscopy at 5°–35°, with the largest diameter of 4.08 ± 1.84 mm and the highest screw insertion rate of 60.42% at 15° and 25°, whereas the diameter and insertion rate are lowest at 45°. The corridor length increases with increasing fluoroscopic angle, and the angle of the corridor axis to the SMP decreases gradually.

**Conclusion:**

The conventional fluoroscopic angle of the pelvic inlet is not suitable for the IAS insertion. The parameters of the IAC vary according to a certain rule under different fluoroscopic angles, so a surgeon can select the appropriate fluoroscopic angle in accordance with the type of fracture and the fracture line angle.

## Introduction

Acetabular fractures are usually intra-articular fractures caused by high-energy injuries. Open reduction and internal fixation have become the standard method for the treatment of unstable acetabular fractures [1]. However, due to the deep location of the acetabulum, the complicated anatomy of the surrounding soft tissues, and the limited intraoperative field of view, it is easy to cause iatrogenic injuries. How to prevent postoperative traumatic arthritis caused by surgical failure to achieve anatomical reduction and fracture re-displacement caused by poor internal fixation are major challenges for traumatologists.

According to Letournel’s acetabular fracture classification [2], the selection of an appropriate surgical approach can significantly improve the stability of fracture fixation, and for complex fracture types, especially in osteoporotic patients, a less invasive single approach is increasingly being promoted because it facilitates rapid postoperative rehabilitation and functional exercise. With the growing popularity of the minimally invasive concept, the channel screw technique has been gradually developed [3, 4]. It has been shown that there are common potential bony channels in the pelvis, which allow for the insertion of corridor screws, and screws such as anterior column screws, posterior column screws, and LC-II screws have been commonly used in fracture types involving the column [5–9].

Culemann et al. [10] improved the screw parallel to the quadrilateral wall proposed by Letournel and introduced the concept of using the infra-acetabular screw (IAS) to connect the anterior and posterior columns by closing the periacetabular fixation frame. The results of two subsequent biomechanical studies showed that the additional insertion of IAS could increase the strength of fracture fixation by 50%, so the use of IAS deserves further investigation [11, 12]. However, there are some controversial studies on infra-acetabular corridor (IAC) parameters. The existing studies show significantly smaller measurements of numerical parameters in Asian people than in European people, and there are some differences in spatial measurements due to the inconsistent choice of reference planes. This makes it difficult for clinicians to refer to existing studies in practice. The purpose of this study is to address the following questions: (1) Is it possible to use the anterior pelvic plane (APP), which is formed by the bilateral anterior superior iliac spine and the pubic symphysis, as the reference plane for pelvic parameter measurements in the patient’s supine position? (2) What are the IAC parameters and screw insertion rates of patients in this region? Are there any differences in IAC parameters between males and females? (3) How do the parameters and screw insertion rates of IAC change under different fluoroscopic angles, and is there a certain rule that can be helpful in clinical operation for fluoroscopy of IAS?

## Methods

### Data collection

All of the procedures comply with the Declaration of Helsinki and relevant Chinese policies. The study protocol was approved by the Ethics Review Committee of Yijishan Hospital Affiliated to Wannan Medical College.

Pelvic data from 187 adult patients who underwent pelvic computed tomography (CT) scan at our hospital from May 2018 to September 2020 are randomly collected for this trial, with the exception of patients with pelvic acetabular fractures, hip dysplasia, pelvic ring deformities, and metal implants in the acetabulum. CT scans are generated by a Philips Brilliance CT 64-Slice scanner (140 kV, 344 mA, and 1 mm reconstructed slice thickness).

### Model reconstruction

Each patient’s pelvic data are imported into Mimics software (Materialise, Leuven, Belgium) in Digital Imaging and Communications in Medicine (DICOM) format, the “CT Bone Segmentation” tool is used, and the bony areas are selected in the section window by the “Add desired seed(s)” command. Then, masks of the pelvis are generated by the software after automatic filling calculation. Finally, the calculate command is used to generate a three-dimensional (3D) model of the pelvis.

### Parameter measurement

**Fig. 1:**
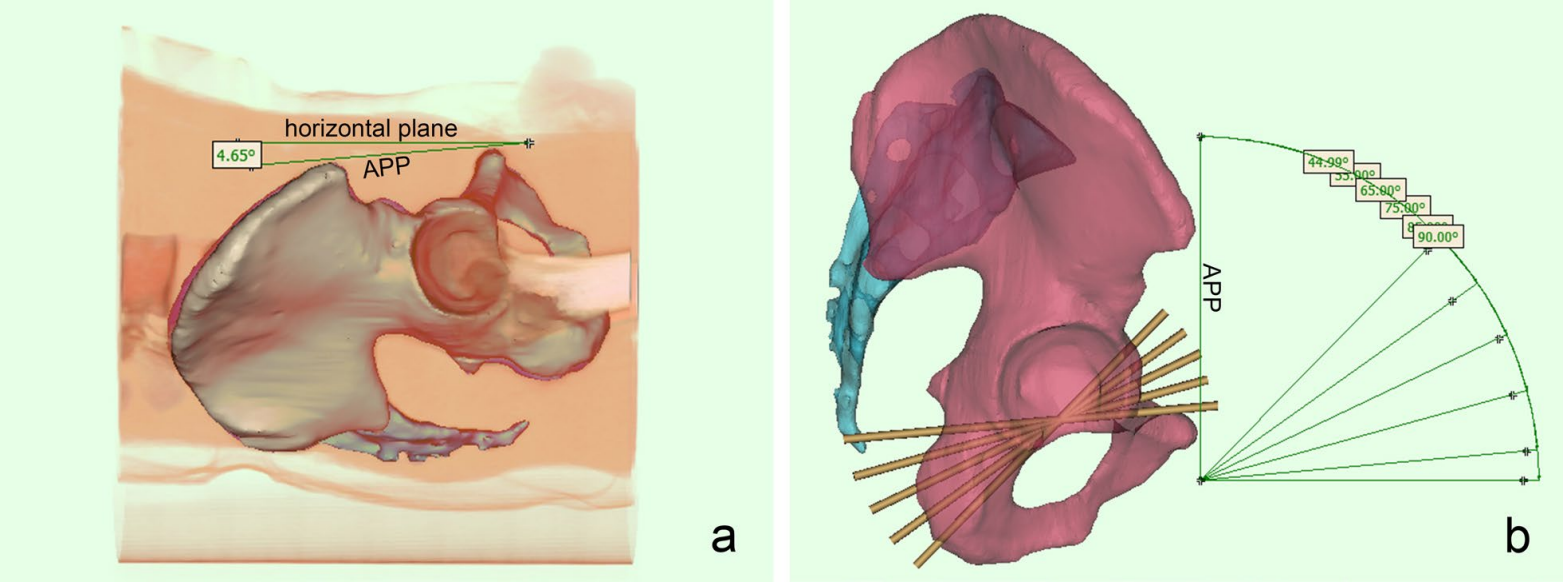
**a** Measure the angle between APP and the horizontal plane of the CT table. **b** Lateral view of pelvic inserted virtual screws in different fluoroscopic angles. APP, anterior pelvic plane

The command, “show the volume rendering”, is used to display the soft tissue of the pelvis and the CT table plate and measure the angle between the APP and the horizontal plane of the CT table (Fig. 1).

**Fig. 2:**
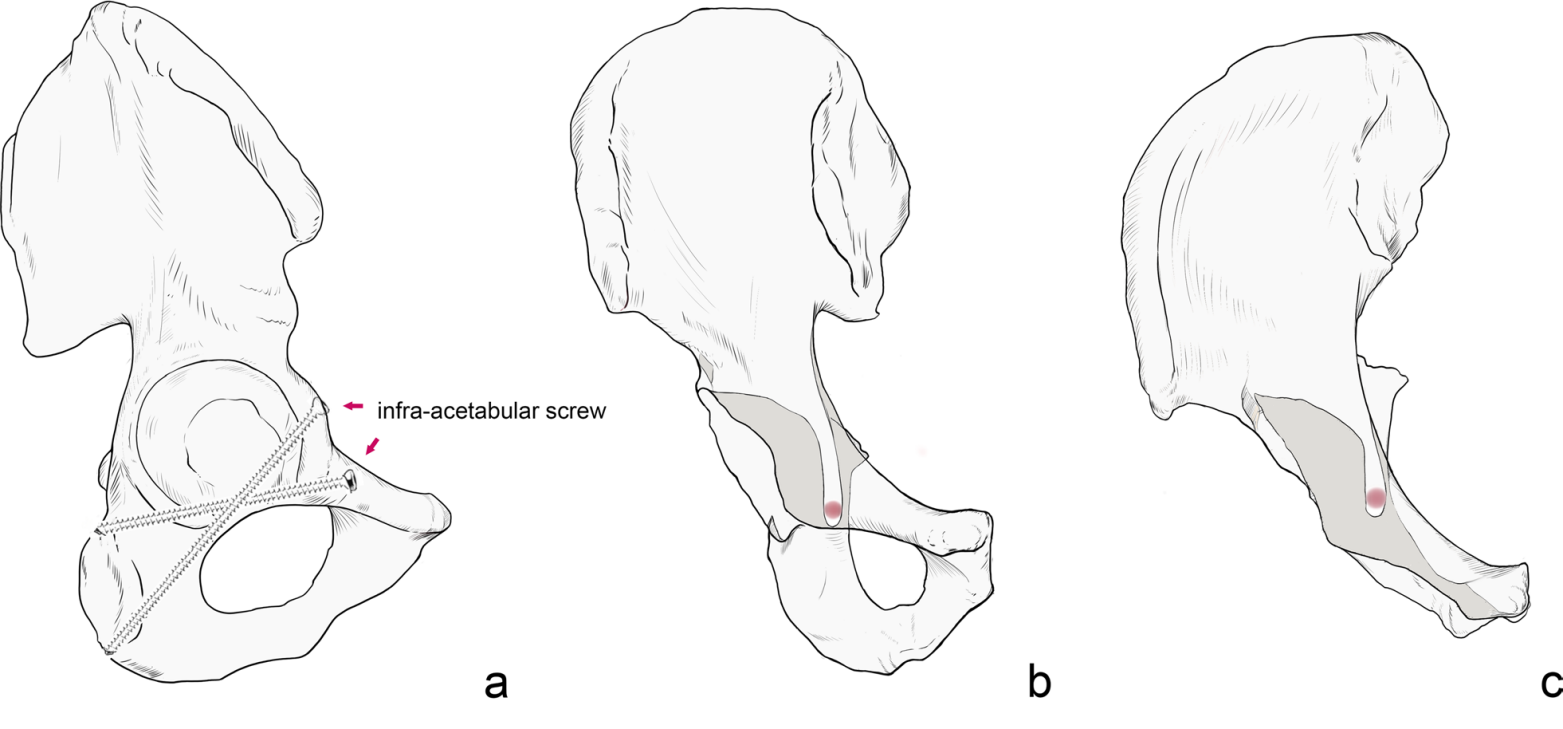
**a** Lateral view of pelvic inserted infra-acetabular screws. **b** Image of the right hemipelvis at a 5° fluoroscopic angle. The red circled areas indicate the axial view of the infra-acetabular screws. **c** Image of the right hemipelvis at a 45° fluoroscopic angle

Firstly, the 3D model of the pelvis is adjusted to the lateral position. We choose the APP as the coronal reference plane, tilt the pelvis forward by 5° (this angle is the fluoroscopic angle), adjust the pelvis to the anterior–posterior position, reduce the transparency of the pelvis, and adjust the fluoroscopic angle along the vertical axis until the largest area of light-colored “U-shaped” area appears in the middle part of the pubic bone on one side, which is the axial projection of the largest corridor under the acetabulum, and the surrounding dark area is the superimposed area of the bone cortex (Fig. 2). We then create a cylinder with a diameter of 1 mm to represent a virtual screw, adjust the screw to the axial perspective, that is, the two ends of the cylinder overlap into a circle, move to the center of the U-shaped area, and gradually increase the diameter of the screw until the screw just does not exceed the boundary of the U-shaped area. By rotating the model, it is repeatedly confirmed that the screw does not break through the cortex. At this time, the screw diameter is recorded as the maximum diameter of the IAC, and then, the maximum diameter of the contralateral side is measured. Each above operation is repeated at the fluoroscopic angles of 15°, 25°, 35°, and 45° (Fig. 1).

**Fig. 3:**
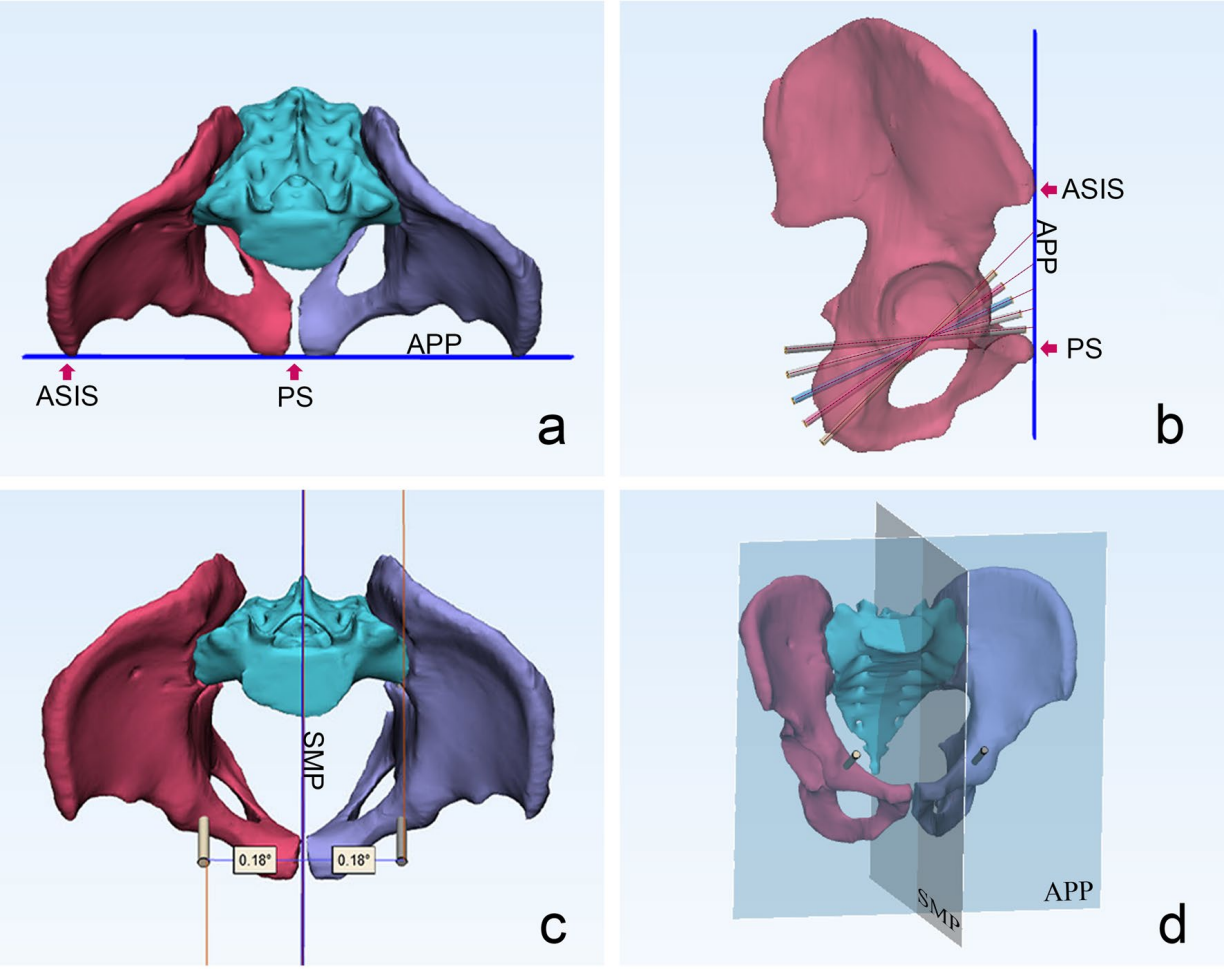
**a** The anterior pelvic plane determined by the anterosuperior iliac spine and the pubic symphysis. The blue line represents the anterior pelvic plane. **b** Lateral view of the pelvis with infra-acetabular screws placement under different fluoroscopic angles. **c** The angle between the screw axis and the sagittal midline plane. **d** The anterior pelvic plane (APP) and sagittal midline plane (SMP). APP, anterior pelvic plane; SMP, sagittal midline plane; ASIS, anterior superior iliac spine; PS, pubic symphysis

The minimum corridor diameter of 3.5 mm is defined as the cutoff while recording the length of the virtual screw in the bone. Using 3-matic software, a sagittal midline plane (SMP) is established to measure the angle between the corridor axis and the SMP (Fig. 3). If the axis direction is from anteromedial to posterolateral, the angle is recorded as a positive value, and if the axis direction is from anterolateral to posteromedial, the angle is recorded as a negative value. All measurement operations are performed by two independent observers, the procedures are repeated twice, and the data are compared to determine the maximum value.

### Statistical analysis

All data are statistically analyzed using SPSS software and expressed in terms of mean and standard deviation (SD). A one-sample *t* test is used to compare whether there is a difference between the tilt angle of the APP and 0° when the patient is in the supine position. An independent sample *t* test is used to compare male and female measurement parameters. The chi-square test is used to compare the screw insertion rates of men and women. The one-way analysis of variance (ANOVA) is used to compare the maximum diameter of the corridor at different fluoroscopic angles, and if there are statistical differences, multiple tests are performed. Pearson correlation analysis is used to correlate epidemiological data (age, height, weight, body mass index (BMI)) with the maximum diameter of the IAC. *P* ≤ 0.05 is considered statistically significant.

## Results

A total of 187 adult patients underwent pelvic CT scan in the Department of Imaging of Yijishan Hospital of Wannan Medical College from May 2018 to September 2020. There were 94 males with an average age of 42 ± 14 years and 93 females with an average age of 45 ± 12 years. The epidemiological data are shown in Table 1. The male and female IAC parameters and screw insertion rates at different fluoroscopic angles are shown in Table 2. The maximum IAC diameter is 4.08 ± 1.84 mm under fluoroscopy at 15° and 25°. The length of the IAC gradually increases with increasing fluoroscopic angle (80.07 ± 5.79 mm → 98.63 ± 7.64 mm), and the angle of the corridor axis in relation to the SMP gradually decreases (5.58 ± 5.52° →  − 0.66 ± 5.52°). Taking 3.5 mm as the cutoff for screw insertion, the maximum screw insertion rate is 60.42% (226/374) at 15° and 25°.

**Table 1:** Epidemiological data of the patient population

	Overall	Male	Female
Number	187	94	93
Age (year)	43 ± 13 (18–65)	42 ± 14 (18–65)	45 ± 12 (18–65)
Height (cm)	164.13 ± 7.39	168.89 ± 4.78	158.31 ± 4.39
Weight (kg)	60.81 ± 13.09	65.75 ± 14.60	55.82 ± 9.00
BMI	22.30 ± 3.18	22.36 ± 3.02	22.24 ± 3.34

BMI, Body mass index

**Table 2:** Measurement results for the ideal position of an infra-acetabular screw under different fluoroscopic angles

	IAD (mm)	IAL (mm)	AIP (°)	IR (%)
5°				
Overall	3.89 ± 1.81	80.07 ± 5.79	5.58 ± 5.52	55.61
Male	4.42 ± 1.80	80.76 ± 5.64	3.40 ± 4.21	68.62
Female	3.35 ± 1.66	75.87 ± 6.04	9.15 ± 5.57	42.47
*t* value	5.96	4.98	− 5.62	*χ*^2^ = 25.89
*P* value	< 0.001	< 0.001	< 0.001	< 0.001
15°				
Overall	4.08 ± 1.84	85.31 ± 7.25	4.61 ± 5.66	60.42
Male	4.60 ± 1.82	87.63 ± 7.15	2.16 ± 4.33	73.94
Female	3.55 ± 1.69	81.94 ± 6.03	8.61 ± 5.30	46.77
*t* value	5.82	5.2	− 9.13	*χ*^2^ = 28.85
*P* value	< 0.001	< 0.001	< 0.001	< 0.001
25°				
Overall	4.08 ± 1.84	92.06 ± 6.95	3.97 ± 5.73	60.42
Male	4.62 ± 1.84	95.02 ± 6.10	1.79 ± 4.31	73.94
Female	3.54 ± 1.68	87.47 ± 5.58	7.57 ± 6.00	46.77
*t* value	6.06	7.15	− 6.38	*χ*^2^ = 28.85
*P* value	< 0.001	< 0.001	< 0.001	< 0.001
35°				
Overall	3.99 ± 1.80	95.64 ± 6.70	1.63 ± 5.76	58.56
Male	4.66 ± 1.82	99.49 ± 5.14	− 0.45 ± 4.56	73.4
Female	3.32 ± 1.50	89.80 ± 4.02	5.14 ± 5.90	43.55
*t* value	7.84	11.91	− 4.4	*χ*^2^ = 34.34
*P* value	< 0.001	< 0.001	< 0.001	< 0.001
45°				
Overall	3.04 ± 2.18	98.63 ± 7.64	− 0.66 ± 4.87	43.58
Male	4.03 ± 2.02	101.96 ± 5.99	− 1.95 ± 3.88	64.89
Female	2.03 ± 1.85	90.10 ± 3.78	3.13 ± 5.54	22.04
*t* value	10.27	5.87	− 4.3	*χ*^2^ = 69.82
*P* value	< 0.001	< 0.001	< 0.001	< 0.001

IAD, diameter of IAC; IAL, length of IAC; AIP, angle between the IAC axis and the sagittal midline plane; if IAC directed to lateral, a value of AIP was positive. IR, insertion rate; χ^2^, chi-squared

In the male sample, the maximum corridor diameter is 4.66 ± 1.82 mm at the fluoroscopic angle of 35°, and the largest screw insertion rate is 73.94% (139/188) at 15° and 25°. The mean length of the IAC gradually increases between 80.76 ± 5.64 mm and 101.96 ± 5.99 mm, and the mean tilt of the corridor axis in relation to the SMP decreases from 3.4 ± 4.21° from anteromedial to posterolateral to − 1.95 ± 3.88° from anterolateral to posteromedial. In the female sample, the maximum corridor diameter is 3.55 ± 1.69 mm under 15° fluoroscopy, and the largest screw insertion rate is 46.77% (87/186) under 15° and 25° fluoroscopy. The mean length of the IAC gradually increases between 75.87 ± 6.04 mm and 90.10 ± 3.78 mm, and the mean tilt of the corridor axis in relation to the SMP decreases from 9.15 ± 5.57° to 3.13 ± 5.54° from anteromedial to posterolateral.

**Fig. 4:**
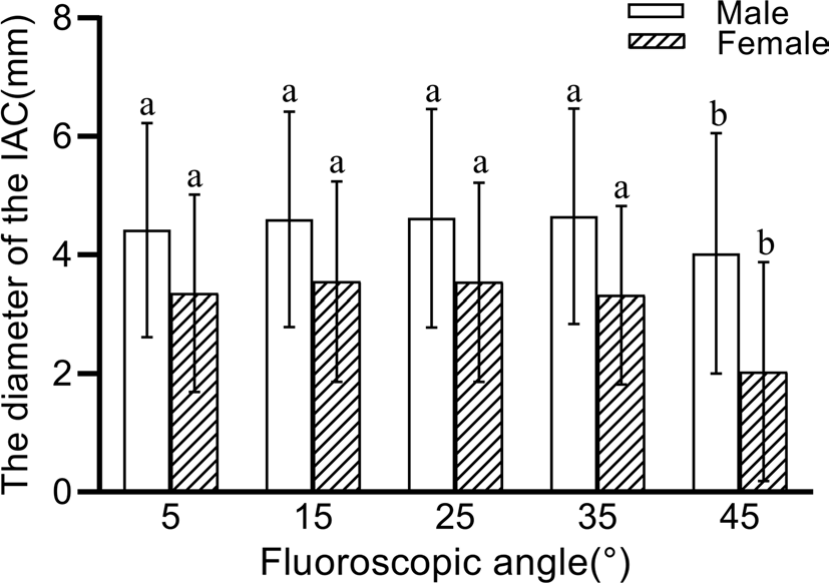
IAC, infra-acetabular corridor. Multiple comparisons of infra-acetabular corridor diameters in men and women at different fluoroscopic angles. The corridor diameter at 45° is significantly different from the results of other groups

The mean angle between the APP and the horizontal plane of the CT table is − 0.43 ± 6.10°, which is not statistically significantly different from 0° according to one-sample *t* test. All parameters of the IAC are significantly different between men and women according to independent sample *t* test, and the rate of screw insertion is also significantly different between men and women at each fluoroscopic angle according to chi-square test (Table 2). One-way ANOVA shows statistically significant differences between the maximum diameter of the IAC at different fluoroscopic angles in men and women. In the male sample, a multiple test shows no significant differences between the maximum diameter of the IAC at 5°, 15°, 25°, and 35°, and a statistically significant difference between the diameter at 45° and the remaining results (Table 3, Fig. 4). In the female sample, the variability of parameters shown by multiple testing is the same as in the male sample (Table 3, Fig. 4). Patients’ epidemiological data show weak correlations with corridor diameter for height and weight, and there was no significant correlation for age and BMI (Table 4).

**Table 3:** Multiple tests of the maximum diameter of the infra-acetabular corridor under different perspective angles

	IAD_5°_	IAD_15°_	IAD_25°_	IAD_35°_	IAD_45°_
Male					
IAD_5°_	×				
IAD _15°_	− 0.17819	×			
IAD _25°_	− 0.19894	− 0.02074	×		
IAD _35°_	− 0.23191	− 0.05372	− 0.03298	×	
IAD _45°_	0.39447*	0.57266*	0.59340*	0.62638*	×
Female					
IAD_5°_	×				
IAD _15°_	− 0.19892	×			
IAD _25°_	− 0.18710	0.01183	×		
IAD _35°_	0.03226	0.23118	0.21935	×	
IAD _45°_	1.32258*	1.52151*	1.50968*	1.29032*	×

IAD, diameter of IAC

*P < 0.05

**Table 4:** Correlation analysis of anthropometric and infra-acetabular corridor diameter

	Age		Height		Weight		BMI	
	*r* value	*P* value	*r* value	*P* value	*r* value	*P* value	*r* value	*P* value
IAD_5°_	0.013	0.865	0.239**	0.001	0.147*	0.044	− 0.012	0.874
IAD _15°_	− 0.009	0.899	0.225**	0.002	0.143	0.051	− 0.003	0.965
IAD _25°_	− 0.010	0.887	0.224**	0.002	0.137	0.062	− 0.005	0.946
IAD _35°_	− 0.082	0.266	0.264**	< 0.001	0.163*	0.026	0.012	0.867
IAD _45°_	− 0.116	0.114	0.354**	< 0.001	0.149*	0.042	− 0.040	0.584

BMI, Body mass index; IAD, diameter of IAC

*P < 0.05; **P < 0.01

## Discussion

Acetabular fracture surgery is one of the most challenging operations in trauma surgery, and because of the acetabulum’s complex anatomy and proximity to important nerves and blood vessels, it is easy to cause iatrogenic injuries during the operation. In order to avoid postoperative traumatic arthritis, anatomical reduction of the fracture ends is particularly important. According to the theory of Judet and Letournel, restoring the stability of the anterior and posterior columns and the integrity of the articular surface are the main objectives of surgical treatment of acetabular fractures [2]. Biomechanical studies have shown that the stability of fracture fixation involving the anterior and posterior columns can be substantially improved by using an additional screw in the infra-acetabular region [11, 12]. However, in practice, due to the extremely limited volume of the bony corridor in the infra-acetabular region, it is difficult to place this screw, so it is rarely used.

The results of this study show no statistical difference between the tilt angle of the APP and 0°, which implies that the APP can be used as the pelvic reference plane for patients in the supine position, independent of the tilt angle of the pelvis and the patient's position, and that a uniform reference plane can be found for different patients according to their anatomical landmarks. The maximum diameter of 4.66 mm is obtained at a fluoroscopic angle of 35° in men, and the maximum diameter of 3.55 mm is obtained at a fluoroscopic angle of 15° in women, with the highest rate of screw insertion for both at 15° and 25° (73.94% vs. 46.77%). When manipulated on the 3D model, it is found that the pelvis is tilted too far at 45°, and part of the pelvic perspective shows the inner surface of the suprapubic ramus for corridor measurement. The traditional 45° pelvic inlet view (i.e., when the patient is lying flat and the radial tube of the C-arm machine tilts 45° toward the patient's head) does not provide a suitable perspective for IAS insertion. The axis of the male corridor is more parallel to the SMP than that of the female corridor, which means that during intraoperative fluoroscopy, the C-arm will need to be rotated more toward the contralateral side to obtain the maximum corridor path in female patients.

The results of a study by Kanezaki et al. [13] showed that in 20% of 80 patients under fluoroscopy with a 25° pelvic inlet view, surgeons were unable to insert IAS because the corridor width was less than 3 mm. The mean diameter of the IAC was 4 mm, and the mean angle between the axis of the corridor and the SMP was 4.7°. Our results are close to the mean diameter of the IAC in this study but are quite different from the results of Gras et al. and Arlt et al., as follows. Gras et al. [14] analyzed 523 pelvises and found that 93% of pelvises had a corridor diameter ≥ 5 mm, with a mean diameter of 7.4 mm, and there was no statistical difference between the 94% male and 90% female screw insertion rates. Arlt et al. [15] studied the morphology of the IAC, and 97% of men and 91% of women had a corridor diameter of 3.5 mm or more. The large variation in the results may be due in large part to the ethnicity of the specimens examined, as Darling et al. [16] showed that Asian women have smaller skeletal dimensions and strength than Caucasian populations. In addition, differences in measurement methods and the choice of reference plane may have a certain effect on the results.

The central part of the IAC has a special biconical anatomical pattern due to the restriction of the acetabular fossa, so unlike the anterior and posterior column screw corridors, where the optimal access path is easier to find, the volume pattern of the IAC is more sensitive to the angle, so the largest screw channel can be found within a certain range [13]. The effect of fluoroscopic angle on the parameters of the IAC has not been described to date, so this study investigated the variation of the parameters of the optimum IAC and the screw insertion rate under different fluoroscopic angles in order to provide guidance for surgical fluoroscopy in clinical practice. IAS insertion is usually performed with the aid of the pelvic inlet view, which is traditionally defined as imaging of the patient in the supine position with the C-arm tube tilted 45° to the cephalad side. However, Ricci et al. [17] recommended the use of a 25° pelvic inlet view based on the results of CT tomographic scans, and the conventional 45° fluoroscopy provides too large an angle of inclination to adequately expose the bony landmarks required for clinical manipulation. Gras et al. [14] showed that the optimal angle between the axis of the corridor to the APP is 54.8°, that is, the fluoroscopy is performed at 35°. Liu et al. [18] found that in contrast with the traditional IAS where the screw-out point is on the ischial tuberosity, placing the screw-out point between the ischial spine and the ischial tuberosity is more suitable for Asian people. At this time, the mean angle between the screw axis and the coronal plane is 75.2°, that is, the fluoroscopy is performed at 15°. The above findings provide a reference for setting the angle grouping in this study.

This study also has obvious limitations. Firstly, all steps are performed on a virtual model, which does not take into account the exposure of the surgical field and the presence of soft tissues around the acetabulum, and the surgical space is extremely limited in practice, which greatly increases the difficulty of screw insertion. Secondly, the screw insertion operation is performed on the complete pelvis model. The clinical situation of fracture reduction largely influences the choice of fixation method. The available bony volume for operation is much smaller than the theoretical value, and screw insertion should be considered only if the reduction is accurate. It is necessary to carry out preoperative planning and fully evaluate the feasibility of screw insertion to avoid iatrogenic injury during the operation.

Research on infra-acetabular screws has led to many recent advancements. When the concept of IAS was first introduced by Culemann, it was considered only applicable to fracture types handled by the ilioinguinal approach (anterior column, anterior column with posterior semi-transverse, T-shape, and double-column fractures), which are driven from the second window, hence the name “second window screws” [10]. Gusic et al. [1] reported retrograde IAS insertion via the posterior K–L approach to fix T-shape with posterior wall fractures and hip dislocation, which first introduced the concept of retrograde IAS. In terms of surgical operation, three-dimensional navigation and electromagnetic navigation techniques have also been applied to screw insertion, and the development of new technologies has undoubtedly helped to shorten the operating time, improve the accuracy of screw insertion, and reduce the radiation dose to the doctor and patient [19, 20]. However, these techniques and hardware facilities are not available in general hospitals, and conventional X-ray fluoroscopy remains the dominant modality to assist with screw insertion, which requires a long learning curve for orthopedic surgeons to master the techniques needed in order to improve the safety and accuracy of access screws.

## Conclusion

In summary, the results of this study suggest that the APP can be used as a reference plane for pelvic measurements. The insertion rate of IAS in this region is low, and screws cannot be placed in approximately 40% of patients. Therefore, this technique cannot be routinely used intraoperatively, and the feasibility of screw insertion must be fully evaluated preoperatively. The traditional 45° pelvic inlet view is not suitable as the perspective angle for IAS insertion; the largest mean diameters of the IAC can be found at fluoroscopic angles of 5°, 15°, 25°, and 35°, and the highest screw insertion rates are at 15° and 25°. In this study, as the perspective angle increased, the corridor length gradually increased, and the angle of the corridor axis to the SMP gradually decreased. During the operation, the appropriate perspective angle can be selected according to the type of fracture and the angle of the fracture line for screw insertion.

## Data Availability

The data in this study are available from the corresponding author upon request.

## Abbreviations

3D: Three-dimensional
CT: Computed tomography
IAS: Infra-acetabular screw
IAC: Infra-acetabular corridor
APP: Anterior pelvic plane
SMP: Sagittal midline plane
ANOVA: Analysis of variance
BMI: Body mass index
DICOM: Digital imaging and communications in medicine
SD: Standard deviation
Mimics: Materialise’s Interactive Medical Image Control System
SPSS: Statistical product and service solutions
